# Transcriptomic Profiling of *Fusarium pseudograminearum* in Response to Carbendazim, Pyraclostrobin, Tebuconazole, and Phenamacril

**DOI:** 10.3390/jof9030334

**Published:** 2023-03-08

**Authors:** Yuan Zhang, Kai He, Xuhao Guo, Jia Jiang, Le Qian, Jianqiang Xu, Zhiping Che, Xiaobo Huang, Shengming Liu

**Affiliations:** 1 Department of Plant Protection, College of Horticulture and Plant Protection, Henan University of Science and Technology, Luoyang 471023, China; 2 National Key Laboratory of Veterinary Public Health Security and School of Veterinary Medicine, China Agricultural University, Beijing 100193, China

**Keywords:** *Fusarium pseudograminearum*, transcriptomic analysis, carbendazim, pyraclostrobin, tebuconazole, phenamacril

## Abstract

*Fusarium pseudograminearum* has been identified as a significant pathogen. It causes Fusarium crown rot (FCR), which occurs in several major wheat-producing areas in China. Chemical control is the primary measure with which to control this disease. In this study, transcriptome sequencing (RNA-Seq) was used to determine the different mechanisms of action of four frequently used fungicides including carbendazim, pyraclostrobin, tebuconazole, and phenamacril on *F. pseudograminearum*. In brief, 381, 1896, 842, and 814 differentially expressed genes (DEGs) were identified under the carbendazim, pyraclostrobin, tebuconazole, and phenamacril treatments, respectively. After the joint analysis, 67 common DEGs were obtained, and further functional analysis showed that the ABC transported pathway was significantly enriched. Moreover, *FPSE_04130* (*FER6)* and *FPSE_11895* (*MDR1*), two important ABC multidrug transporter genes whose expression levels simultaneously increased, were mined under the different treatments, which unambiguously demonstrated the common effects. In addition, Mfuzz clustering analysis and WGCNA analysis revealed that the core DEGs are involved in several critical pathways in each of the four treatment groups. Taken together, these genes may play a crucial function in the mechanisms of *F. pseudograminearum*‘s response to the fungicides stress.

## 1. Introduction

*Fusarium pseudograminearum* was first detected in Queensland, Australia, in 1951. It is responsible for crown rot in wheat, and it causes severe economic losses of more than 1 billion dollars every year [1]. In 2011, this pathogen was reported for the first time in the wheat-producing area of Henan Province, showing an incidence rate of more than 70% [2]. As straw is perennially returned to fields, it results in the accumulation of fungal sources, and thus, the disease has been continuously worsening and spreading. *F. pseudograminearum* has become an important pathogen affecting wheat production in the Yellow River and Huaihe River wheat regions of China [3,4]. The fungus not only causes wheat crown rot but also a variety of serious crop diseases, including wheat head and seedling blight, as well as maize stalk rot and cob rot [5,6,7].

In recent years, information on the fungicide resistance of *F. graminearum*, *F. asiaticum,* and *F. pseudograminearum*, with regard to benzimidazoles (carbendazim and thiophanate-Methyl), triazoles (tebuconazole, hexaconazole, and Epoxiconazole), cyanoacrylates (phenamacril), and strobilurin (pyraclostrobin and azoxystrobin) fungicides, has been frequently reported [8,9,10,11]. Benzimidazole fungicides, such as carbendazim, are a large class of fungicides that was developed in the 1960s and 1970s. They mainly act on the β-tubulin of pathogenic fungi, thus preventing the formation of spindle filaments, thereby interfering with its nuclear division [12,13,14]. Triazole fungicides, such as tebuconazole, which are characterized by a broad bactericidal spectrum and high activity, were developed in the 1970s and 1980s. This type of fungicide inhibits the activity of 14α-demethylase by combining the heme-iron active center of lanosterol 14α-demethylase and the nitrogen atom on the heterocycle; this hinders the synthesis of ergosterol, and thus, this type of fungicide eventually becomes bactericidal [15]. The cyanoacrylate fungicide phenamacril, which targets myosin-5 in the pathogenic fungi, exhibits a high specific activity against *Fusarium* spp. [16,17]. The quinol oxidation inhibitors (QoIs) fungicides, such as pyraclostrobin, are a new class of fungicides derived from the natural product strobilurin; these fungicides were developed in the late 1990s [10]. They are also characterized by a broad spectrum and high efficiency, they are environmentally friendly, and they do not target other organisms. This type of fungicide inhibits the respiration of mitochondria by preventing the Qo site binding in the cytochrome *bc1* complex; thus, the mitochondria cannot produce and provide the energy required for cell metabolism, and this eventually leads to cell death.

By utilizing Next-Generation Sequencing (NGS) platforms, RNA-Seq can efficiently and accurately present the transcript information of a specific material. This technique has become a ubiquitous tool in molecular biology research, and it has greatly advanced the understanding of genome function over the past years. For instance, a transcriptome analysis by Zhang et al. [18] demonstrated that thymol accumulated the reactive oxygen species (ROS) of *Fusarium oxysporum*, and it disrupted the integrity of the cell wall and cell membrane, thus explaining the underlying fungicidal mechanism. Zheng et al. identified a series of DEGs that may be related to resistance regulation and the fungicidal activity of phenamacril with acomparative transcriptome analysis of resistant and sensitive *F. oxysporum* strains after phenamacril treatment [19]. Wang et al. explored the fungistatic effect of thymol on *F. graminearum* via transcriptomic analysis; the analysis indicated that the gluconeogenesis/glycolysis pathway may be simultaneously involved in the growth of *F. graminearum* hyphae and the production of deoxynivalenol (DON) when treated with thymol [20].

As far as we know, little research has been conducted on the fungicidal activity of the four abovementioned fungicides and their effects on the pathogen *F. pseudograminearum*, which poses a great threat to the quality and safety of crops; thus, this is the first article to dissect the gene expression patterns of *F. pseudograminearum* for these fungicides. This article aims to examine the different mechanisms of action via RNA-Seq, with the intention of filling the gap in the literature. These results can provide insights into the mechanism that drives the fungicidal activity against *F. pseudograminearum*, and therefore, it may provide the foundation for developing novel fungicides to control the crown rot of wheat. 

## 2. Materials and Methods

### 2.1. Fungal Strains, Culture Media, and Fungicides

The *F. pseudograminearum* strain, XX1809, that was used in this study was collected from crown base disease samples of a major wheat-producing area, which is located in Henan Province, China. The strain was routinely incubated on potato sucrose agar (PSA, 200 g of potato, 16 g of agar, and 20 g of sucrose per liter of distilled water) plates at 25 °C. Technical-grade carbendazim (98%) was provided by the Jiangsu Rotam Chemistry Co., Ltd, Jiangsu, China. Technical-grade phenamacril (99%) was kindly supplied by Jiangsu Pesticide Research Institute Co., Ltd, Jiangsu, China. Technical-grade salicylhydroxamic acid (99%), pyraclostrobin (97.5%), and tebuconazole (97%) were kindly provided by Nanjing Agricultural University. Carbendazim was dissolved in 0.1 M of hydrochloric acid. The remaining fungicides were dissolved in methanol to obtain a 10 mg/mL stock solution and stored at 4 °C. 

### 2.2. Sensitivity Determination of Mycelial Growth

In the study, the final concentrations were 0, 0.1, 0.3, 0.5, 0.7, and 0.9 µg/mL for the carbendazim treatment group; 0, 0.03125, 0.0625, 0.125, 0.25, and 0.5 µg/mL for the pyraclostrobin treatment group; 0, 0.05, 0.1, 0.2, 0.4, 0.6, and 0.8 µg/mL for the tebuconazole treatment group; 0, 0.625, 0.125, 0.25, 0.5, and 1 µg/mL for the phenamacril treatment group. These concentrations were used to test how sensitive the strain XX1809 was to these fungicides. Salicylhydroxamic acid (SHAM), a specific alternative oxidase inhibitor, was added the pyraclostrobin treatment group with the concentration of 100 μg/mL. Mycelial discs (Ø 5 mm) of the pathogen were removed from the edge of the 3-day-old colony with a sterile punch, and they were placed in the center of the PSA plates containing corresponding concentrations of the fungicides described above. After incubating for three days, at 25 °C, in the dark, the colony diameter was measured by using the cross method. The 50% effective concentration (EC_50_) values were calculated in accordance with the regressing percentage growth inhibition against the log of fungicide concentration [21]. In this study, the four different treatment groups were named as follows: carbendazim was named CAR, tebuconazole was named TEB, phenamacril was named PHE, pyraclostrobin was named PYR, and the control group was named CK.

### 2.3. RNA Extraction, Library Construction, and Sequencing

Ten mycelial plugs (Ø 5 mm) from the edge of the 3-day-old colony of *F. pseudograminearum* strain XX1809 were transferred into a flask containing 100 mL of 3% mung bean soup media (MS, mung bean, 30 g per liter of distilled water) and shaken (150 rpm, 25 °C, 12 h photoperiod). A total of 6 flasks were used. Seven days later, the broth was filtered by two layers of lens tissue, the conidia were harvested, and the filtrate was centrifuged at 5000 rpm until the solution was colorless. The number of conidia was calculated with a hemocytometer under a microscope and diluted to 1 × 10^5^/mL; then, the conidia suspension (1 mL) of the strain XX1809 (1 × 10^5^) was inoculated in 100 mL of Yeast Extract Peptone Dextrose (YEPD) medium (0.3% of yeast extract, 2% of dextrose, and 1% of peptone per liter of distilled water), incubated for 24 h, shaken at 150 rpm, at 25 °C, and kept under dark conditions. The carbendazim, tebuconazole, phenamacril, and pyraclostrobin treatments, at their corresponding EC_50_ concentrations, were added to the flasks; pathogen samples that were not treated with fungicides were the control samples. After being incubated in the same environment for 7 days, the fresh mycelium from 15 samples was centrifuged and collected with three biological replicates per treatment group for subsequent transcriptome sequencing. The total RNA was extracted using a Trizol reagent (TAKARA, Kusatsu, Japan) in accordance with the manufacturers’ instructions. The concentration and purity of the obtained RNA were then measured with a Nanodrop 2000 spectrophotometer (Thermo Scientific, Shanghai, China). Moreover, the RNA integrity and RIN values were measured via agarose gel electrophoresis and Agilent2100, respectively. Then, using 1 μg of total RNA, the cDNA library was constructed with the TruSeqTM RNA sample preparation Kit from Illumina (San Diego, CA, USA), followed by sequencing with the Illumina NovaSeq 6000 platform comprising a paired-end 150 bp strategy (San Diego, CA, USA).

### 2.4. Transcriptome Data Processing and the Identification of DEGs

First, quality control processing of raw sequencing data was performed using fastp (v 0.19.5) in order to remove low-quality reads and adapter-containing reads. This enabled the filtering of reads whose unknown nucleotide content exceeded 10% and low-quality base content exceeded 50% (Q value < 20). The clean data were then compared with the reference genome of *F. pseudograminearum* (GCA_000303195.2 https://www.ncbi.nlm.nih.gov/genome/14399?genome_assembly_id=293398 (accessed on 3 July 2022) using HISAT2 (v2.2.1) for the subsequent analysis. StringTie (v2.1.2) software was used to assemble and count the read numbers that were mapped to each gene. The normalized expression quantification results were transformed using a TPM method (Transcripts Per Kilobase of exon model per million mapped reads). To gain additional insights into the coding genes, all protein sequences of the genome were aligned against the six public functional databases (GO, Gene Ontology, http://geneontology.org/ (accessed on 5 July 2022); KEGG, Kyoto Encyclopedia of Genes and Genomes, http://www.kegg.jp/kegg/kegg1.html (accessed on 5 July 2022); NCBI_NR, NCBI non-redundant protein library, ftp://ftp.ncbi.nlm.nih.gov/blast/db/ (accessed on 5 July 2022); Swiss-Prot, http://web.expasy.org/docs/swiss-prot_guideline.html (accessed on 5 July 2022); PFAM, Protein Families Database, http://pfam.xfam.org/ (accessed on 5 July 2022); EggNOG, Evolutionary genealogy of genes: non-supervised orthologous groups, http://eggnog5.embl.de/#/app/home (accessed on 5 July 2022)) via a BLASTX search with an E-value threshold of ≤1 × 10^−5^. If available, the hit among the characterized genes with the lowest E-scores was screened and functionally annotated. Afterward, differential expression analysis of groups within the four comparison groups was performed using the ‘DESeq2′ package (v 1.37.5). Differentially expressed genes (DEGs) were identified as a gene expression change greater than 2 times and false discovery rate-adjust *p* < 0.05 (|Log2FC ≥ 1| and FDR < 0.05).

### 2.5. Gene Ontology and KEGG Pathway Enrichment Analysis

The GO functional enrichment analysis of DEGs was performed using the software Goatools (https://github.com/tanghaibao/goatools (accessed on 7 July 2022)), and the KEGG enrichment analysis of DEGs was performed using KOBAS (http://kobas.cbi.pk-u.edu.cn/home.do (accessed on 7 July 2022)). Results that exhibited a *p*-value less than 0.05 were considered to be significantly enriched. 

### 2.6. Fuzzy C-Means Clustering and WGCNA Analysis 

To better investigate the gene expression pattern variations among the different treatments, all DEGs from the four comparison groups were combined in order to examine the relationship between transcript expression and the different treatments. This was achieved via the following two methods: fuzzy C-Means clustering analysis using the ‘Mfuzz’ package and Weight gene co-expression network analysis (WGCNA) using the ‘WGCNA’ package in R. 

Regarding the WGCNA analysis, a co-expression network was constructed with a beta value of 9, which was used in the analysis. Then, genes were clustered into branches of highly expressed genes, and featured modules were detected using the dynamic tree-cutting method, which was further classified and clustered with mergeCutHeight = 0.25 and minModuleSize = 30. The differential groups in the study were used as traits for the WGCNA analysis [22,23], and the group-specific modules were identified based on the module–trait relationship (the correlation between the eigengene and traits). Modules with an absolute correlation coefficient > 0.55 and *p*-value < 0.05 were identified as group-specific modules. Protein–protein interaction networks with a confidence score ≥0.4 were constructed using the STRING (Search Tool for Retrieval of Interacting Genes/Proteins) database (http://string-db.org/ (accessed on 10 July 2022)). Hub genes are those that show the most connections and correlations in the network as indicated by the most accurate maximal clique centrality (MCC) algorithm of cytoHubba, which are further visualized using Cytoscape (v 3.9.1, https://cytoscape.org/ (accessed on 10 July 2022)). For genes in each important module, KEGG pathway enrichment analyses were conducted to analyze the biological function of modules. 

### 2.7. Validation of Selected DEGs via RT-qPCR Analysis

To verify the reliability of the data obtained by RNA-Seq, nine genes were randomly selected for RT-qPCR analysis. The total RNA extracted from the samples were reverse-transcribed to cDNA using the PrimeScript RT kit and gDNA Eraser (TAKARA, Kusatsu, Japan). The RT-qPCR reaction was performed on a Bio-Rad (CFX96 Touch) using TB Green^®^ Premix Ex Taq (TAKARA, Kusatsu, Japan), in accordance with the instructions provided. Primers were designed using primer 5.0, and they were synthesized using Sangon Biotechnology (Shanghai, China) (Table S1). The expression data were normalized using *FPSE_11980* (Elongation factor 1-alpha, *TEF1a*) as an internal control. The experiments were repeated three times with three replicates for each sample. The relative expression levels of genes were calculated using the 2^−ΔΔCT^ method [20], and the standard deviation was calculated from the three biological replicates.

## 3. Results

### 3.1. Sensitivity Determination 

**Figure 1:**
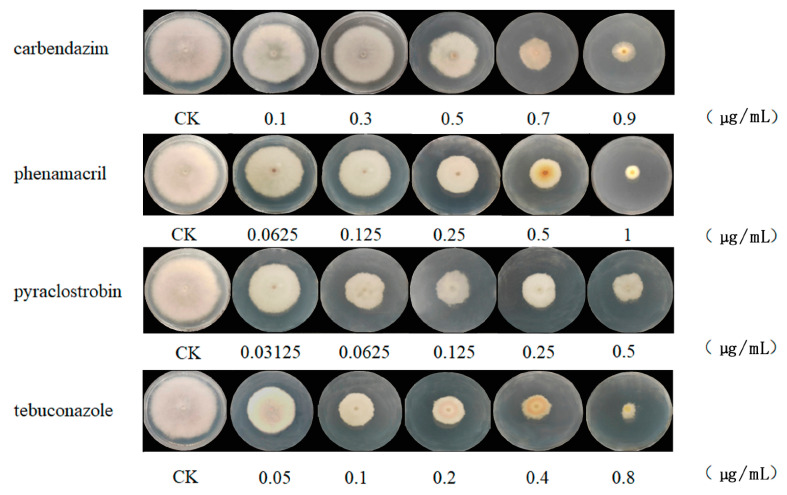
Sensitivity of *F. pseudograminearum* to pyraclostrobin, carbendazim, phenamacril, and tebuconazole was determined by the mycelial growth rate.

The fungicidal effects of carbendazim, pyraclostrobin, tebuconazole, and phenamacril on the mecelial growth of *F. pseudograminearum* XX1809 were determined using the fungal growth rate method. The results showed that the EC_50_ values of XX1809, obtained when the pathogen was working against carbendazim, pyraclostrobin, tebuconazole, and phenamacril, were 0.5 μg/mL, 0.15 μg/mL, 0.12 μg/mL, and 0.22 μg/mL, respectively (Figure 1, Table S2). Based on the EC_50_ values of XX1809, which were obtained when the pathogen was working against the four fungicides, XX1809 was treated with the EC_50_ of each agent in YEPD medium and subjected to further examinations.

### 3.2. General Features of the Transcriptome Data

RNA sequencing generated a total of 108.98 G of clean data for all 15 samples after they were subjected to the quality control process. Approximately 7.26 G of reads for each sample were used for subsequent analyses. For all libraries, the proportion of Q20 exceeded 97.94%; Q30 did not exceed 94.47%; on average, the GC content was 51.59%. After these results were aligned, the percentage of sequence reads, for the CK, PYR, CAR, PHE, and TEB groups, was mapped onto the *F. pseudograminearum* reference genome. The percentage ranges were as follows: 87.68 to 91.51%, 87.62 to 90.28%, 86.85 to 89.63%, 81.22 to 87.17%, and 87.77 to 91.29%, respectively (Table S3). This provides a global indicator for sequencing accuracy.

**Figure 2:**
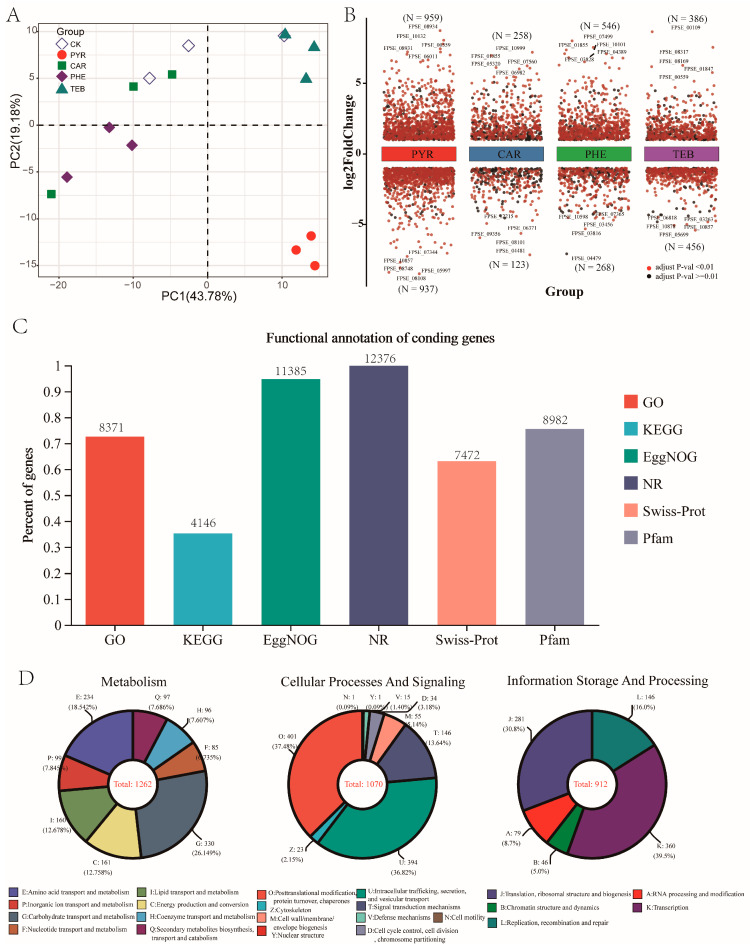
Overview of the *F. pseudograminearum* transcriptome data. (**A**) Principal component analysis (PCA) of all RNA-Seq data (PC1 (43.78%) was the first principal component, and PC2 (19.18%) was the second principal component); (**B**) scatter plot showing the number of DEGs across the four comparison groups, where the color black represents a significant difference (*p*-adjust value > 0.01) and the color red represents a highly significant difference (*p*-adjust value < 0.01); (**C**) bar plot showing the percentage and number of annotation genes in the six public databases (GO, KEGG, EggNOG, NR, Swiss-Prot, Pfam); (**D**) the number of known annotated genes involved in metabolism, cellular processes and signaling, and information storage and processing in the EggNOG database.

For the gene expression patterns, the overall distribution plot of the TPM normalized expression levels, for all samples, is displayed in Figure S1A. In addition, the correlation coefficients in all treatment groups had values of more than 0.89, as compared with the CK group (Figure S1B). The most obvious separation was observed between the CK and PYR group with a correlation coefficient of 0.93–0.94, whereas the correlation coefficient with 0.93–0.97 was observed between the CK group and CAR and PHE treatment groups. Consistent with this, the principal components analysis (PCA) also displayed a trend that showed that the gene expression of the PYR groups deviated from the gene expression of the other groups (Figure 2A). These data revealed that different treatments have diverse effects on the growth of *F. pseudograminearum*.

### 3.3. Functional Annotation and DEGs Analysis

In total, 12,397 coding genes have been identified and published with regard to the genome of *F. pseudograminearum*, while the vast majority of genes were hypothetical proteins without defined annotation information. Given that, we performed a functional comparison with six public functional databases via a BLASTX algorithm (E-value ≤ 1 × 10^−5^); the relative expression levels of all samples and the annotation information of all genes are given in Table S4. Out of a total of 12,397 coding genes, 12,376 (99.8% of the total coding genes) had a match in the NR database and 8371 (70.4%), 4146 (33.4%), 11,385 (91.8%), 7472 (60.3%), and 8982 (72.4%) genes showed significant similarities with sequences from the GO, KEGG, EggNOG, Swiss-Prot, and Pfam databases (Figure 2C). Furthermore, among the 11,386 identified genes in the EggNOG database, 1262, 1070, and 912 genes were annotated with known functional classifications pertaining to metabolism, cellular processes and signaling, and information storage and processing, respectively (Figure 2D). 

Differential expression analysis revealed that pyraclostrobin, carbendazim, phenamacril, and tebuconazole induced significant alterations in the global gene transcriptional profile of *F. pseudograminearum*. Compared with the CK group, 1896 (959 up- and 937 down-regulated), 381 (258 up- and 123 down-regulated), 814 (546 up- and 268 down-regulated), and 842 genes (386 up- and 456 down-regulated) were differentially expressed in the PYR, CAR, PHE, and TEB groups, respectively (Figure 2B). It is evident that the number of DEGs was highest in the PYR treatment group and lowest in the CAR group, whereas the number was similar in the PHE and TEB treatment groups. Moreover, the top five most significantly up- and down-regulated genes in each comparison group were labeled in the plot. Among these labeled up-regulated genes in the PYR, CAR, and PHE groups, several transport-related genes were identified according to Swiss-Prot annotation, including *FPSE_06011* (ZEB2-regulated ABC transporter 1, *ZAR1*) in the PYR group, *FPSE_10999* (MFS efflux transporter, *ALCA*) in the CAR group, and *FPSE_04389* (uncharacterized transporter) in the PHE group. For the TEB treatment group, the over-expression of three encoding steroid biosynthesis-related genes including *FPSE_00109* (sterol 14-alpha-demethylase, *CYP51*), *FPSE_08317* (sterol 24-C-methyltransferase, *SMT1*), and *FPSE_01847* (sterol 22-desaturase, *ERG5*) were found. These results primarily revealed the functional similarities and dissimilarities between the four fungicides.

### 3.4. The Common Genes Affected by the Different Fungicides

**Figure 3:**
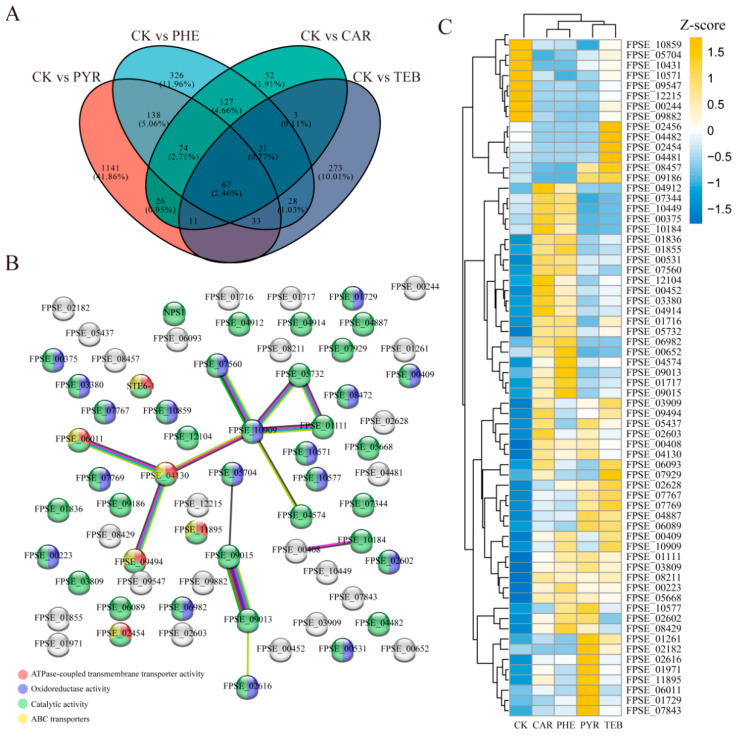
The common genes affected by the four fungicides. (**A**) The Venn diagram shows the number and percentage of the common or unique differential genes in each comparison group (CK versus PYR, CAR, PHE, and TEB); (**B**) protein–protein interaction (PPI) networks were constructed based on the 67 common genes. The edges in the network represent the interactions between two genes, and the different colors represent enriched features; (**C**) the heatmap of 67 common gene expression level changes in the five groups. The colors in the heatmap represent the gene expression values that were normalized using the Z-score method; yellow represents up-regulation and blue represents down-regulation.

The common genes were further screened to evaluate the joint effects that they had on *F. pseudograminearum* when subjected to the different treatments. All the comparison groups shared 67 DEGs, while 1141, 326, 52, and 273 DEGs were only significantly expressed in the PYR, PHE, CAR, and TEB treatment groups, respectively (Figure 3A). The PPI network was then constructed via the STRING database in order to show the mutual relationship between these 67 DEGs; this network was mainly contributed by the data concerning the interactions between the 14 nodes and 12 edges (Figure 3B). Notably, two genes that are related to carbohydrate transport and metabolism, *FPSE_09015* (glucosamine-6-phosphate isomerase 1, *GNPDA1)* and *FPSE_09013* (N-acetylglucosamine-6-phosphate deacetylase, *AMDHD2*), were found to have a strong correlation with one another; they had a combined score that was close to 1. Moreover, the corresponding functional analysis showing the three GO terms, including ATPase-coupled transmembrane transporter activity, oxidoreductase activity, catalytic activity, and the ATP-binding cassette (ABC) transporters pathway, indicated significant enrichment levels; this was mainly shown via the highlighted gene in the network. Furthermore, the gene expression level changes and the detailed annotation for the 67 DEGs were also displayed (Figure 3C and Table S5). Most DEGs (55 out of 67 genes) showed a consistent up-regulated or down-regulated trend compared with the CK group. Moreover, 16 genes were annotated simultaneously via the EggNOG database, with 14 up-regulated and 2 down-regulated genes (Table S5). Among these genes, three genes associated with ABC transport, *FPSE_04130* (multidrug resistance protein, *FER6)*, *FPSE_06011* (ZEB2-regulated ABC transporter 1, *ZAR1*), and *FPSE_11895* (ABC multidrug transporter, *MDR1*), were simultaneously significantly up-regulated. Notably, another gene encoding *ZAR1* (*FPSE_09494*), without the EggNOG annotation, was found to have a strong link with *FPSE_06011* and *FPSE_04130*, and they also have a similar expression trend. Taken together, these results revealed several key genes that are related to the transport function and facilitate an understanding of the common effects of the four fungicides with regard to *F. pseudograminearum*. 

### 3.5. Functional Alterations Affected by the Different Fungicides

**Figure 4:**
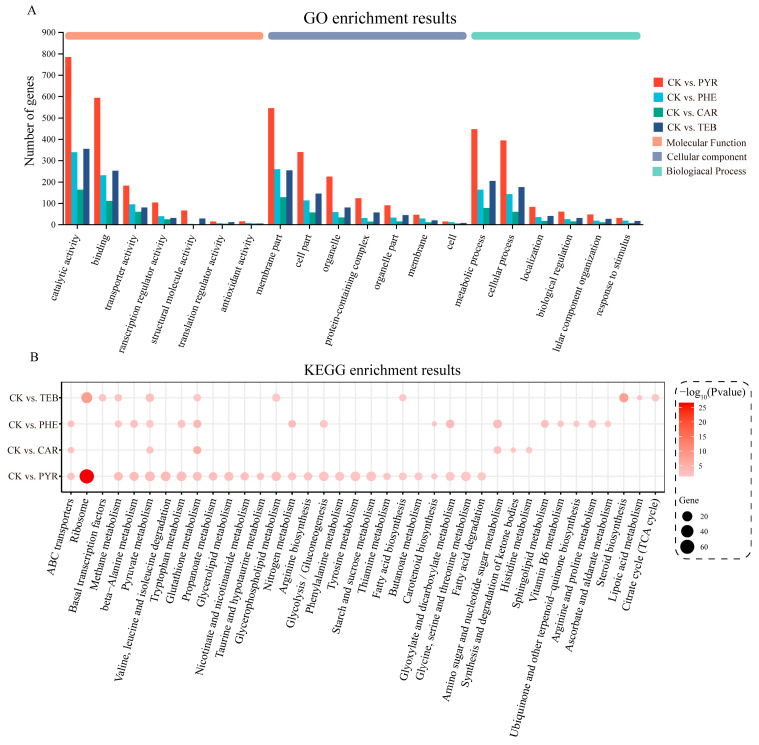
GO term and KEGG pathway enrichment analysis. (**A**) The bar plot of the GO function enrichment analysis targeting the Biological Process (BP), Cell Component (CC), and Molecular Function (MF); (**B**) the bubble plot of the KEGG pathway enrichment analysis. The size represents the number of genes, and the color represents the *p*-value.

To explore the biological characteristics of these DEGs in greater detail, GO and KEGG enrichment analyses were conducted in order to identify the main functional features. For the GO enrichment analysis, the results showed that the top three Molecular Functions (MFs) were catalytic activity, binding, and transporter activity; the top three Cellular Components (CCs) were the membrane part, cell part, and organelle; the top three Biological Processes (BPs) were the metabolic process, cellular process, and localization (Figure 4A). The KEGG pathway analysis also showed a significant alteration in the 38 pathways (Figure 4B). Of these, the vast majority of pathways (35/38) were subject to metabolism-related changes. Compared with the control group, the four treatment groups simultaneously significantly enriched two pathways, which were ‘pyruvate metabolism’ and ‘glutathione metabolism’ pathways. The ‘ABC transporters’ pathway, which is the only pathway associated with membrane transport, was present in the three groups except for TEB treatment. Similarly with the results of GO enrichment, PYR and CAR treatment had the most and least significant effect, relative to other groups, respectively.

**Figure 5:**
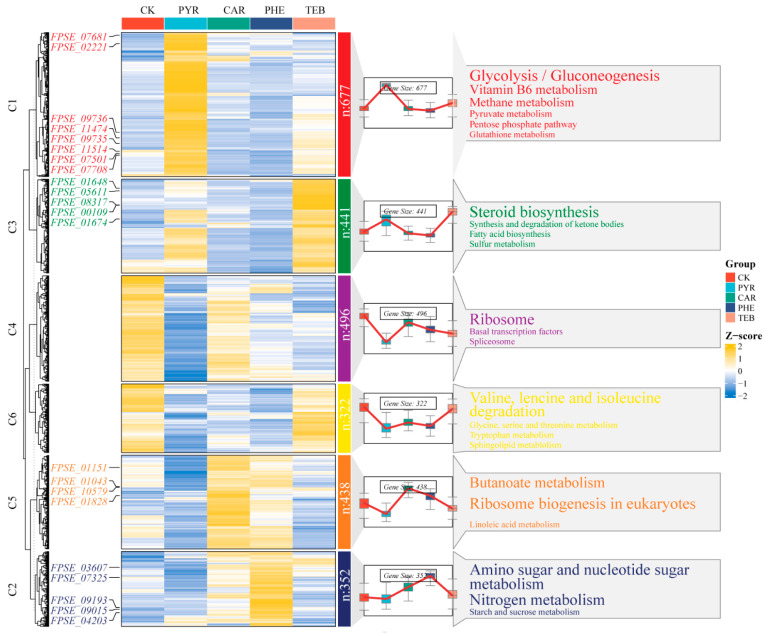
Clustering trend analysis based on the fuzzy C-mean algorithm. The gene clustering heatmap and trend diagram illustrate the different expression patterns, the corresponding KEGG enrichment results are displayed on the right-hand side, and the representative genes of each cluster are shown on the left-hand side of the heatmap.

To gain more precise insights into the dynamic gene expression changes of DEGs when subjected to different treatments, we performed the clustering trend analysis using the fuzzy c-mean algorithms that were based on a combination of the 2726 DEGs from the four comparison groups. The total number of DEGs were clearly grouped into six clusters (C1-C6), and they demonstrated the varied response patterns that emerged when the interactions between *F. pseudograminearum* and the four fungicides were taking place. As shown in Figure 5, each heatmap and dynamic line plot present the corresponding change patterns, with regard to gene expression in the individual cluster. The potential KEGG pathways (*p* < 0.05) were also shown on the right-hand side of Figure 5. Moreover, the key DEGs that are labeled on the left-hand side of Figure 5 are also shown in Table 1. We noticed that most of the 677 genes in C1, 352 of the genes in C2, 441 of the genes in C3, and 428 of the genes in C5 exhibited higher expression in the PYR, PHE, TEB, and CAR groups, respectively, whereas there exists a decreased gene expression trend in C4 and C6, as compared with the CK group. 

In C1, the carbohydrates, energy, cofactors, and vitamins associated with metabolism were significantly enriched, such as glycolysis/gluconeogenesis, pyruvate metabolism, vitamin B6 metabolism, and the pentose phosphate pathway. *FPSE_11474* (NADP-dependent alcohol dehydrogenase 7, *ADH7*), *FPSE_11514* (enolase, *ENO*), *FPSE_09735* (pyruvate decarboxylase, *PDC*), *FPSE_02221* (hexokinase, *HK*), *FPSE_07501* (pyruvate kinase, *PK*), *FPSE_11514* (acetyl-coenzyme A synthetase, *ACS*), and *FPSE_07708* (transketolase, *TKLIA*) may play important roles in these carbon pathways. Amino sugar and nucleotide sugar metabolism, and nitrogen metabolism were significantly enriched in C2. *FPSE_03607* (glutamine synthase, *GLNA*) and *FPSE_07325* (putative formamidase, *E3.5.1.49*), which are involved in energy metabolism, were significantly up-regulated. Steroid biosynthesis, fatty acid biosynthesis, and sulfur metabolism were significantly enriched in C3. In particular, *FPSE_00109* (sterol 14-alpha-demethylase, *CYP51*) and *FPSE_08317* (sterol 24-C-methyltransferase, *SMT1*) were up-regulated significantly, by more than 200-fold. The genes in C5 were mainly involved in Butanoate metabolism. Of these, *FPSE_01151* (3-ketoacid coenzyme A transferase, *OXCT*) and *FPSE_01828* (nuclear GTP-binding protein) were significantly enriched. Additionally, regarding the other two clusters (C4 and C6), relative to the CK group, containing ribosome and valine, leucine and isoleucine degradation caused the most significant enrichment, respectively. More details concerning the expression level and annotation of other genes that are associated with the enriched pathways in each cluster are presented in Table S6. The over-expressed key DEGs in each cluster were considered to be important regulated factors when subjected to the different treatments.

### 3.6. Identification of Hub Genes and Network Construction

**Figure 6:**
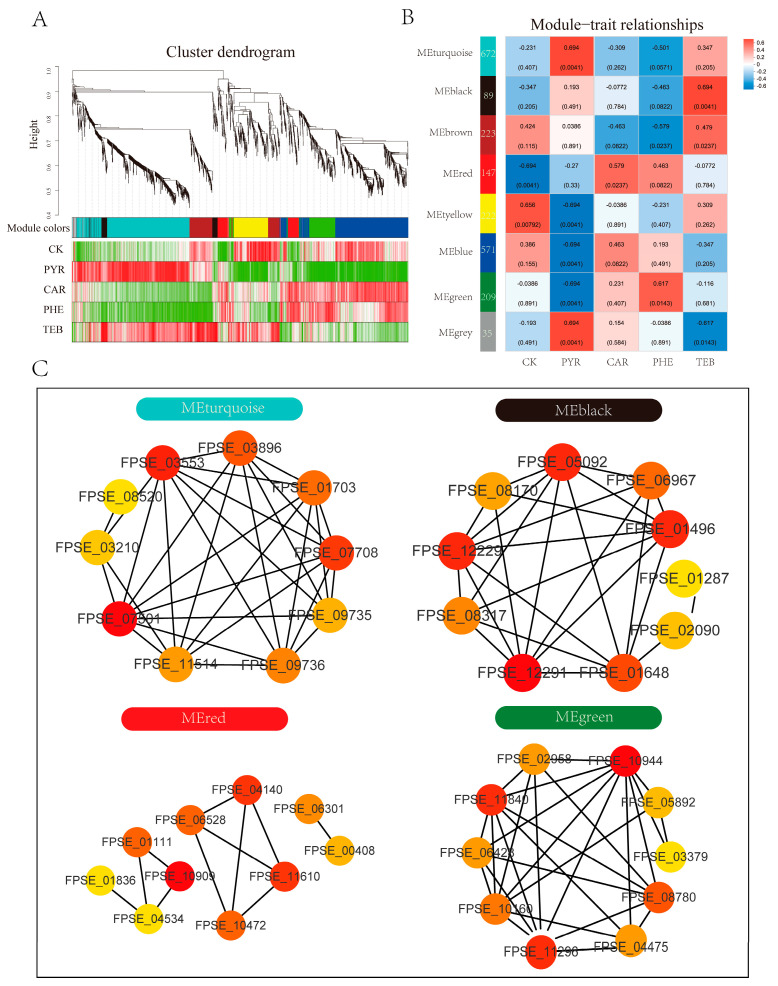
WGCNA analysis and network construction. (**A**) Clustering dendrogram of genes with dissimilarities based on the topological overlap, together with the assigned merged module colors; (**B**) the heatmap of the module–trait relationship. The abscissa represents the group, and the ordinate represents the module plotted with the module eigenvalues. The color bar on the right represents the level of correlation from low (blue) to high (red), and the number of DEGs in each module is shown; (**C**) the top 10 MCC score-ranked genes in the four modules, including turquoise, black, red, and green, were calculated using cytoHubba and visualized with Cytoscape.

To investigate the hub genes of network regulation, taking the response of *F. pseudograminearum* to the different fungicides into consideration, the DEGs mentioned above were used to preform WGCNA analysis, which, therefore, led to the identification of eight WGCNA modules (Figure 6A). Of these modules, an analysis of the module–trait relationships indicated that the module ‘turquoise’ with 672 genes, module ‘red’ with 147 genes, module ‘green’ with 209 genes, and module ‘black’ with 89 genes were significantly positively correlated with the PYR, CAR, PHE, and TEB groups (r ≥ 0.55 and *p* < 0.05), respectively (Figure 6B); therefore, these hub genes in the four crucial modules were thought to play important roles in each of the distinct treatment groups. Functional enrichment analysis was first performed to determine their role in metabolic pathways (Figure S2). Interestingly, we found that the KEGG enrichment results of the turquoise, green, black, and red modules identified by WGCNA analysis were similar to those of C1, C2, C3, and C5, as identified by Mfuzz method, which was controlled by the high-expression gene during the PYR, PHE, TEB, and CAR treatments, respectively. The genes in the turquoise module were mainly enriched in glycolysis/gluconeogenesis, pyruvate metabolism, and glutathione metabolism. Steroid biosynthesis and sulfur metabolism were significantly enriched in the black module. In the red module, butanoate metabolism and lysine degradation were primarily enriched. Amino sugar and nucleotide sugar metabolism, and nitrogen metabolism were significantly enriched in the green module.

Moreover, for the four key modules, cytoHubba was utilized in order to identify the top 10 essential genes (hub genes) with the most accurate MCC algorithm, which could be served as the core regulated factors in the network and visualized by Cytoscape (Figure 6C and Table S7). The hub genes of the turquoise module were mostly related to the central carbon metabolisms, such as *FPSE_07501* (pyruvate kinase, *PK*), *FPSE_03553* (pyruvate dehydrogenase, *PDHA*), *FPSE_07708* (transaldolase, *TKL*), *FPSE_11514* (enolase, *ENO*), and *FPSE_09735* (pyruvate decarboxylase, *PDC*). In the red modules, *FPSE_10909* (glutathione reductase, *GSR*), *FPSE_11610* (heat shock 70 kDa protein, *HSPA1s*), and *FPSE_04140* (hsp70 nucleotide exchange factor, *HSPBP1*) contribute the most. Especially in the green module, nine of the top ten hub genes were significantly down-regulated in the PYR group, and *FPSE_10944* (heat shock protein 90, *HSP90A*), *FPSE_11840* (mitochondrial protein import protein, *MAS5*), and *FPSE_11296* (dnaJ-related protein, *RSP1*) significantly increased in the PHE group. In the black module, *FPSE_12291* (delta (7)-sterol 5(6)-desaturase, *SC5DL*), *FPSE_05092* (squalene synthase, ERG6), and *FPSE_01496* (eburicol 14-alpha-demethylase, *CYP51*) contributed the most, and the other hub genes had the same expression pattern. Notably, certain overlapped results could be found in the identified core genes from WGCNA analysis and the identified genes via the Mufzz method (Table S6), which further verified the reliability of the results.

### 3.7. Real-Time PCR Validation

**Figure 7:**
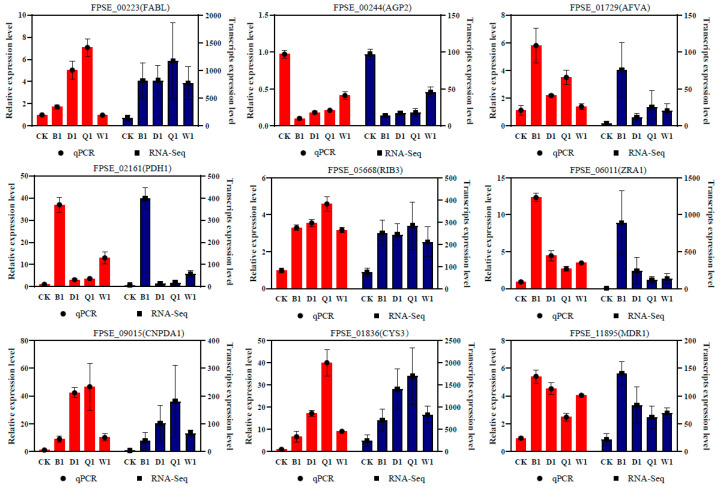
Validation of the expression of the candidate genes during infection by quantitative Real-time RT-qPCR analysis. Nine DEGs were randomly selected for RT-qPCR. These genes included *FPSE_00223* (enoyl-[acyl-carrier-protein] reductase, *FABL*), *FPSE_00244* (general amino acid permease, *AGP2*), *FPSE_01729* (NADPH dehydrogenase, *AFVA*), *FPSE_02161* (phytoene desaturase, *PDH1*), *FPSE_05668* (3,4-dihydroxy-2-butanone 4-phosphate synthase, *RIB3*), *FPSE_06011* (ZEB2-regulated ABC transporter 1, *ZRA1*), *FPSE_09015* (glucosamine-6-phosphate isomerase 1, *GNPDA1*), *FPSE_01836* (cystathionine gamma-lyase, *CYS3*), and *FPSE_11895* (ABC multidrug transporter, *MDR1*). The bars represent the standard deviations (±SD) of the mean.

Nine genes were randomly selected to validate the accuracy of the RNA-Seq data by RT-qPCR. The comparative bar plot showed that the relative expression level changes of the corresponding genes in the different treatment groups were similar using the two methods, which confirmed the reliability of the expression profile based on the RNA-Seq data (Figure 7).

## 4. Discussion

In recent years, the fungicidal activities of carbendazim, pyraclostrobin, tebuconazole, and phenamacril have been extensively reported in most pathogens [12,13,16,17,24,25,26,27]. However, few studies have examined the activity of these fungicides against *F. pseudograminearum*, which is regarded as the most damaging pathogen causing crown rot in wheat, and it is a pathogen that has been newly identified in Henan province, China [4]. However, due to the low ratio of gene annotation on the pathogen, functional investigations have been limited. In this study, the comprehensive and specific data for carbendazim, pyraclostrobin, tebuconazole, and phenamacril, concerning their effects on the transcriptome of *F. pseudograminearum,* were obtained using the RNA-Seq approach, which will be helpful for understanding the different mechanisms of action of the fungicides targeting *F. pseudograminearum*. Moreover, these data may also provide more references for subsequent scientific research concerning the organism.

The ABC transporter has served as the most common protein family with multiple roles, which mediate many crucial physiological processes, such as metabolic detoxification, internal and external signal transduction, lipid homeostasis, pathogen defense, and antigen progressive presentation [28,29,30,31]. In recent years, the distribution, classification, aspects of fungal resistance, and fungal pathogenicity for ABC transporters in numerous plant pathogens, including *Magnaporthe oryzae*, *Botrytis cinereal,* and *Fusarium graminearum* [32,33,34,35,36,37], have been receiving increased levels of attention. For instance, it has been shown that both insertional mutations and gene deletions in the ABC gene can lead to a significant reduction in the ability of the fungus to infect the rice or barley epidermis [38]. Three ABC transports related to multidrug resistance in *Botrytis cinereal*, Bcatrab [34,39], BcatrD, and BcatrK [40], exhibited varied sensitivities to different drugs after the knockout. Meanwhile, the exporters of the reported ABC transporters were able to play similar functions; this occurs in almost all organisms [41]. In addition, the functional characterization of the 60 *F. graminearum* ABC proteins, as shown by Yin et al., revealed that the deletion mutants of *FgArb1* microorganisms have increased susceptibility to oxidative stress, defective cell wall integrity, and the production of DON is reduced [42]. In our study, a joint analysis of the four fungicides showed that certain features, including transmembrane transporter activity in GO terms and ABC transporters in KEGG pathways, were significantly enriched. In particular, two genes associated with the multidrug transporters, *FPSE_04130* (*FER6*) and *FPSE_11895* (*MDR1*), simultaneously exhibited increased expression levels after interacting with the four fungicides. Taken together, these results indicated that the two key genes might play a major role in the metabolic transport of the four fungicides that work against *F. pseudograminearum*.

As well as evaluating the joint effects, we also focused on exploring the key regulators that target individual treatments by combining the Mfuzz and WGCNA methods. Pyraclostrobin and tebuconazole, the two fungicides that have more DEGs, play a central role in glycolysis/gluconeogenesis and steroid biosynthesis, respectively. Several key genes underwent significant changes under PYR treatment, thus enabling them to play a vital role in the central carbon pathways of *F. pseudograminearum*. These include changes caused by the pyruvate kinase (*FPSE_07501*), decarboxylase (*FPSE_09735*), enolase (*FPSE_11514*), transketolase (*FPSE_03896*), and phosphoenolpyruvate synthase (*FPSE_109736*). In a previous study, pyruvate kinase, a key enzyme in the glycolytic pathway, was discovered as a potent and novel fungicidal target of the fungicide YZK-C22, and it exhibited specific antifungal activity [43]. Wang et al. also reported that pyruvate decarboxylases (PDCs)-mediated plant metabolic pathways contribute to plant resistance to bacterial wilt [44]. For instance, Arabidopsis and tomatoes responded to *Ralstonia solanacearum* infection with an increase in PDC activity, whereas plants with insufficient PDC activity were more susceptible to bacterial wilt. Furthermore, enolase is a highly conserved and widespread glycolytic pathway for key enzymes, and it is found in all types of living organisms. It also plays an important role in pathogen invasion [45]. Another previous study showed that once transketolase activity is inhibited, the Calvin cycle of transketolase-dependent substrates and products, starch and sucrose synthesis, the manganate pathway, and phenylpropanoid metabolism are also all inhibited, which could be used as a target site for new fungicides [46]. Moreover, tebuconazole is a DMI class of fungicides belonging to the class of ergosterol Sterol Biosynthesis Inhibitors (SBIs). Ergosterol exists in most fungi, and it is an essential component of the cell membrane; it regulates critical biological functions, such as the fluidity and permeability of the cell membrane, which is a crucial factor for cell survival [47]. In our study, levels of *FPSE_00109* and *FPSE_01496* encoding sterol 14 alpha-demethylase (*CYP51*) and *FPSE_08317* encoding Sterol 24-C-methyltransferase (*SMT1*) were obviously elevated in the TEB group. *CYP51* has been regarded as an important target for antifungal drug development, and the inhibition of *CYP51* is effective for limiting fungal growth [48]. Sterol C-24 methyltransferase was also reported to be able to catalyze the methylation reaction of yeast sterols to generate coprosterol [49,50].

As for carbendazim and phenamacril, several genes related to heat shock 70 kDa protein were highly expressed in the two groups, such as *FPSE_11610* and *FPSE_04475* in the PHE group and *FPSE_04140* in the CAR group. Heat shock proteins (Hsps) have been reported to be related to many general stress responses, rather than thermal stress responses, and they play essential roles in cell signaling transduction, as well as cell cycle and apoptosis regulation. In a study of *Candida albicans*, several heat shock proteins were discovered and they were proven to have the potential to be a promising target for antifungal effects [51,52]. Additionally, several proteins involved in ribosome biogenesis and protein assembly showed higher expression levels in CAR groups, such as *FPSE_01828* (*NUG1*) and *FPSE_00408* (*RRB1*). It is known that ribosome biogenesis is a very tightly regulated process, and it is more closely linked to other cellular activities such as growth and division [53]. In the PHE group, *FPSE_09193* encoding chitin synthase 8 was significantly up-regulated. Chitin has been regarded as an indispensable structural component in insects and fungi but not in plants and mammals; therefore, chitin synthase has become one of the potential targets for the synthesis of efficient, safe, and eco-friendly pesticides [54]. A previous study showed that the deletion of gene FgChs7, which encodes a chitin synthase, led to reduced mycelial growth, virulence, or increased sensitivity to various stresses in *F. graminearum* [55]. Together, these results from previous studies, combined with our data, uncover the several key pathways and genes that underlie the *F. pseudograminearum* response to different fungicides.

To the best of our knowledge, the present study is the first to report the relationship between the four fungicides (PYR, PHE, TEB, and CAR) and the transcriptome alteration of *F. pseudograminearum*. A comprehensive assessment of the transcriptome data obtained in the study was illustrated in detail. However, the functions of the acquired key genes that regulate the related key metabolic pathways will need to be further studied with other tools such as RNAi or the gene knockout strategy, which offer new prospects for the potential targets of therapeutic intervention in fungal diseases.

## 5. Conclusions

Overall, the present study set out to comprehensively reveal the resistance mechanism for the *F. pseudograminearum* response to the pyraclostrobin, tebuconazole, phenamacril, and carbendazim fungicides. A joint analysis identified the two ABC multidrug transporter-related genes (*FPSE_04130* and *FPSE_11895*), which were significantly elevated in all four treatments. In addition to the common effect that they had on the pathogen, several critical pathways were found significantly enriched under the varying treatments. Among that, glycolysis/gluconeogenesis, steroid biosynthesis, amino sugar and nucleotide sugar metabolism, and butanoate metabolism play a leading role for the above four treatments, respectively. Moreover, Mfuzz and WGCNA analyses further identified the high expression or hub genes involved in these important pathways for each fungicide, which provide a new explanation of the interaction between fungi and fungicides. Future studies should focus on the functional validation of the key genes responsible for the different fungicides seeking novel antifungal targets.

## Figures and Tables

**Table 1:** The key DEGs that are involved in responding to the four fungicides in the different clustering trend.

Gene_ID	Name	Swiss-Port Description	Log2 Foldchange (CK vs. Treatment)				Cluster
			PYR	CAR	PHE	TEB	
FPSE_11474	ADH7	NADP-dependent alcohol dehydrogenase 7	4.72	-	-	-	1
FPSE_11514	ENO	Enolase	1.29	-	−2.04	-	1
FPSE_09735	PDC	Pyruvate decarboxylase	3.83	-	-	-	1
FPSE_07501	PK	Pyruvate kinase	1.61	−1.32	−1.40	-	1
FPSE_07681	ACS	Acetyl-coenzyme A synthetase	3.09	-	1.84	-	1
FPSE_02221	HK	Hexokinase	3.55	-	-	-	1
FPSE_09736	ppsA	Phosphoenolpyruvate synthase	3.69	-	-	2.25	1
FPSE_07708	TKLlA,	Transaldolase	1.26	-	-	-	1
FPSE_09193	CHS8	Chitin synthase 8	-	-	1.64	-	2
FPSE_09015	GNPDA1	Glucosamine-6-phosphate isomerase 1	3.72	5.02	6.05	4.11	2
FPSE_03607	GLNA	Glutamine synthetase	-	-	1.55	-	2
FPSE_07325	E3.5.1.49	Putative formamidase	−3.41	-	1.15	-	2
FPSE_04203	TPSA	alpha-trehalose-phosphate synthase	-	-	1.63	-	2
FPSE_01648	TM7SF2	Delta (14)-sterol reductase	-	-	-	1.19	3
FPSE_00109	CYP51	Sterol 14-alpha-demethylase	-	-	-	8.66	3
FPSE_08317	SMT1	Sterol 24-C-methyltransferase	-	-	-	7.55	3
FPSE_05611	PDHB	Pyruvate dehydrogenase E1 component subunit beta	1.11	-	-	1.61	3
FPSE_01674	ACACA	Acetyl-CoA carboxylase	1.98	-	-	1.88	3
FPSE_01151	OXCT	3-ketoacid coenzyme A transferase	−1.61	1.17	-	-	5
FPSE_01828	NUG1,	Nuclear GTP-binding protein	-	1.88	-	-	5
FPSE_01043	NMD3	nonsense-mediated mRNA decay protein 3	-	1.35	-	-	5
FPSE_10579	TGL4	Lipase 4	-	1.61	-	-	5

Note: ‘-’ represents no significant change in the comparison group.

## Data Availability

The raw sequence data reported in this paper have been deposited in the Genome Sequence Archive in the National Genomics Data Center, China National Center for Bioinformation/Beijing Institute of Genomics, Chinese Academy of Sciences (GSA: CRA009390) that are publicly accessible at https://ngdc.cncb.ac.cn/gsa (accessed on 10 January 2023) [56,57].

## Supplementary Materials

The following supporting information can be downloaded at: https://www.mdpi.com/article/10.3390/jof9030334/s1 Figure S1: Analysis of the relationship between samples; (A) Distribution of gene expression levels (TPM-normalized). (B) Correlation heatmap of gene expression in control and other four drug treatment groups. The blue color blue represents the low correlation, and the red color represents the high correlation; Figure S2: Top 10 KEGG enrichment analysis for the identified WGCNA modules generated by hierarchical clustering of WGCNA. The Rich Factor is the ratio of the number of genes in the particular module to the number of genes annotated in the feature. The greater the Rich factor, the greater the degree of enrichment; Table S1: Primers used for RT-qPCR analysis; Table S2: Sensitivity of *F. pseudograminearum* strain XX1809 to the four fungicides; Table S3: Summary of transcriptome sequencing datasets; Table S4: The normalized expression level (TPM) and annotation information of all identified genes in transcriptomic analysis; Table S5: The gene expression level change and the detailed annotation of the 67 shared DEGs; Table S6: The KEGG enrichment results for the Mfuzz clustering analysis; Table S7: Top 10 hub genes in the network interactions of the four major modules ranked by MCC method.
