# Supplementary figures and images for: Transcriptomic Profiling of Fusarium pseudograminearum in Response to Carbendazim, Pyraclostrobin, Tebuconazole, and Phenamacril
Source: J Fungi (Basel). 2023 Mar 8;9(3):334. doi: 10.3390/jof9030334 (PMC10057576; doi:10.3390/jof9030334)

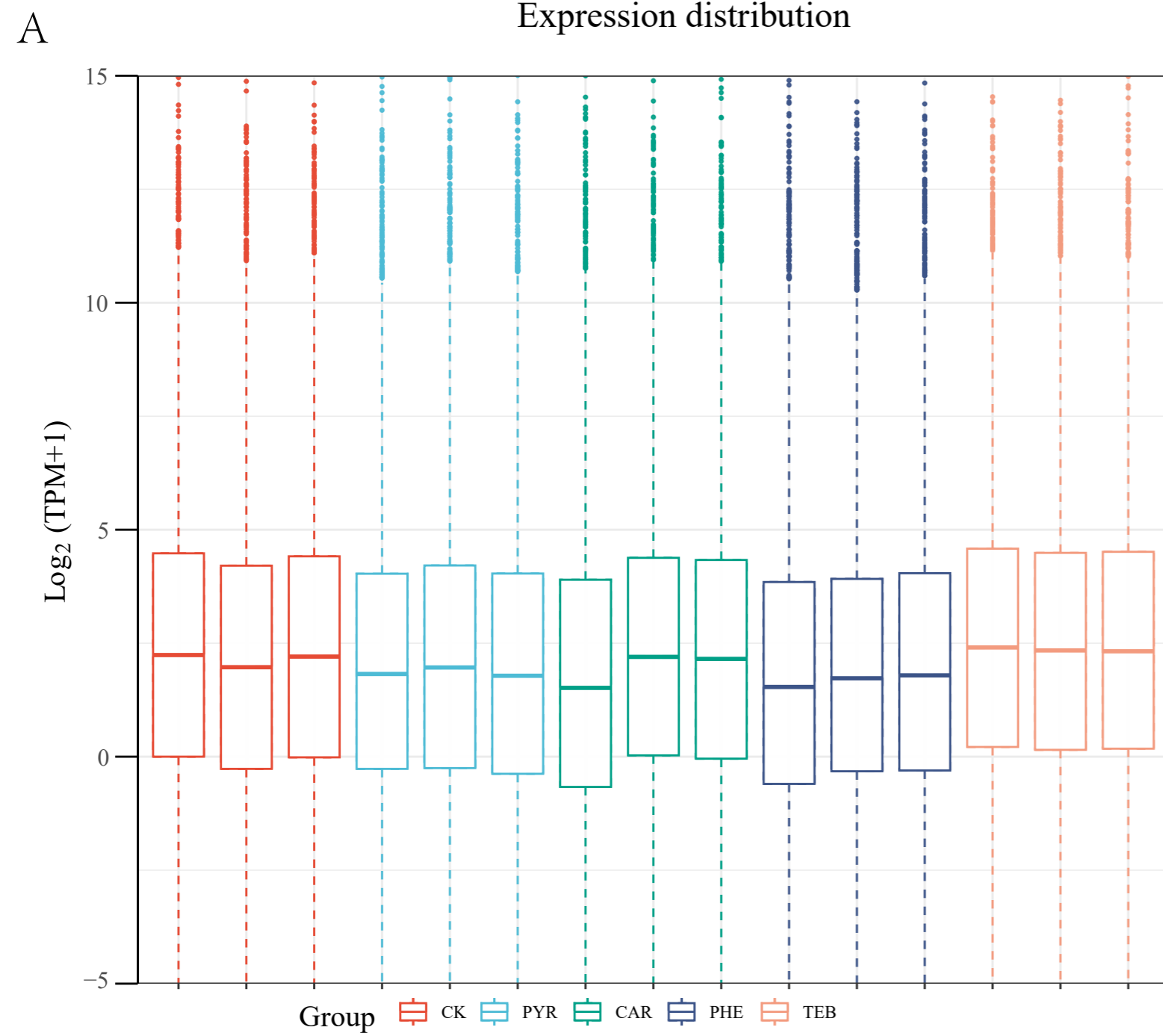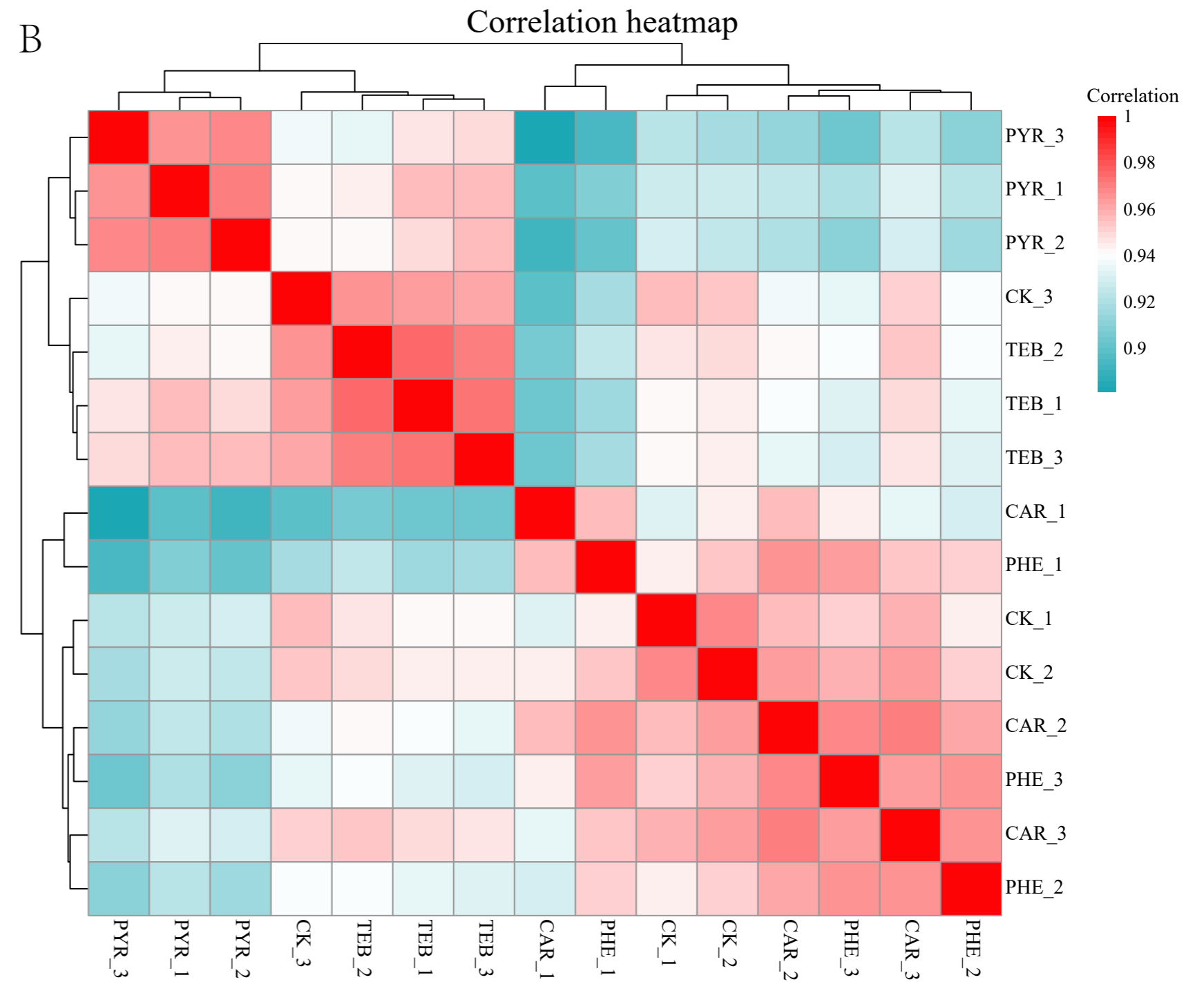

Supplement: Supplementary file 1 [file jof-09-00334-s001.zip › Fig S1.pdf]

# Kegg Enrichment Analysis

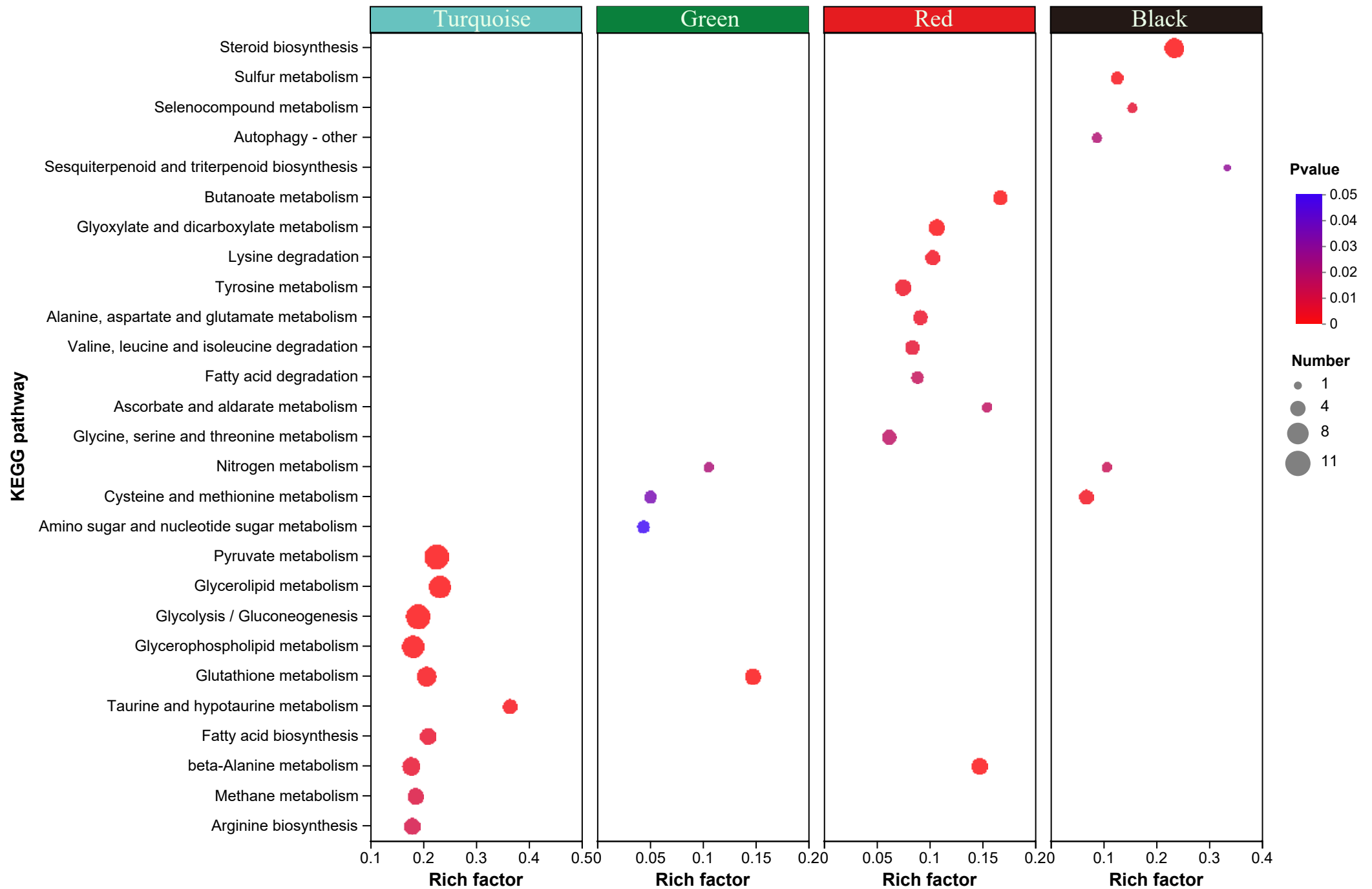

Supplement: Supplementary file 1 [file jof-09-00334-s001.zip › Fig S2.pdf]
